# Transactions of the Ohio State Medical Society

**Published:** 1861

**Authors:** 


TRANSACTIONS OF THE OHIO STATE MEDICAL SOCIETY; 1860.—Columbus: Follett, Foster & Co.

We are in receipt of this handsome octavo volume of nearly 300 pp., substantially bound in cloth, and, in typographical appearance, well worthy its valuable contents—the proceedings of the fifteenth annual meeting of the Ohio State Society, held at White Sulphur Springs, in June last.

Among the papers published, we notice an interesting report from the Committee on *Cannabis Indica*, by R. B. McMeans, M. D., of Sandusky.

The report of the Committee on Medical Literature—E. B. Stevens, M. D., Cincinnati—is peculiarly interesting and valuable, as furnishing a pretty thorough *resume* of the condition of this important interest. From it we glean, that of Medical journals, devoted to the interests of legitimate medicine, we have three weeklies, eighteen monthlies, eleven bi-monthlies, and three quarterlies; of which number—thirty-five in all —New York and Ohio, each, publish five; Pennsylvania and Georgia, each, four; Missouri, three; Louisiana, Illinois, Tennessee, California and Kentucky, each, two; and Massachusetts, North Carolina, South Carolina and Virginia, each, one. This does not include, of course, those publications devoted to specialities, as the *American Journal of Insanity*, that able quarterly,—nor the numerous magazines of pharmacy, chemistry, dental science, etc.; still less the "abundant parasitic growth of periodical literature engaged in promulgating the vagaries of eclecticism, homœopathy, phrenology, spiritualism and 'rational medicine,' a sort of illegitimate creation, voluminous and frothy."

The number of these regular publications has been remarkably uniform, we learn, for a series of years; though, of course, changes occur from time to time. Our writer appreciatingly places Medical Journalism *first*, "because of its pervading influence, because it demands the most prompt attention of medical men, and because it is the medium through which nearly everything new and valuable first comes before us."

The following assertions we are already prepared to endorse most fully: The medical journals of this country are a working, practical institution; poorly fed and poorly clothed, to be sure; but they never refuse their full share in the great mass of drudgery, which the machinery of our profession requires to be performed. * * * * * The glory enjoyed by medical journalists, is, for the most part, regarded as ample compensation for the pains and penalties of mind, body and purse, which are incident to so honorable a position.

Dr. Wright's elaborate paper on the *Effects of Chloroform on the Intellectual Processes*, will well repay perusal; while Dr. Metz's report on *Diseases of the Eye*, and Dr. Gundry's report on *Insanity*, are papers of marked ability,—the latter particularly rich in valuable statistics, soundest philosophy, and enlightened study and research.

